# Oxidative stress and nitric oxide signaling related biomarkers in patients with pulmonary hypertension: a case control study

**DOI:** 10.1186/s12890-015-0045-8

**Published:** 2015-05-02

**Authors:** Shuai Zhang, Ting Yang, Xiaomao Xu, Meng Wang, Linye Zhong, Yuanhua Yang, Zhenguo Zhai, Fei Xiao, Chen Wang

**Affiliations:** Department of Pulmonary and Critical Care Medicine, Beijing Institute of Respiratory Medicine, Beijing Chao-Yang Hospital, Capital Medical University, Beijing, People’s Republic of China; Beijing Key Laboratory of Respiratory and Pulmonary Circulation Disorders, Beijing, People’s Republic of China; Department of Pulmonary and Critical Care Medicine, Beijing Hospital, Beijing, People’s Republic of China; National Clinical Research Center of Respiratory Diseases, Beijing, People’s Republic of China; Department of Laboratory Medicine, Beijing Hospital, Beijing, People’s Republic of China; Department of Cell Biology, Institute of Geriatrics, Beijing Hospital, Beijing, People’s Republic of China; Department of Respiratory Medicine, Capital Medical University, Beijing, People’s Republic of China; China-Japan Friendship Hospital, Beijing, People’s Republic of China

**Keywords:** Oxidative stress, Nitric oxide, Pulmonary hypertension, Biomarkers

## Abstract

**Background:**

Oxidative stress (OS) and reduced nitric oxide (NO) bioavailability contribute to the pathogenesis of pulmonary hypertension (PH). Whether there are associations between OS and NO signaling biomarkers and whether these biomarkers are associated with the severity of PH remain unclear.

**Methods:**

Blood samples were collected from 35 healthy controls and 35 patients with pulmonary arterial hypertension (PAH, n = 12) or chronic thromboembolic pulmonary hypertension (CTEPH, n = 23). The mean pulmonary artery pressure (mPAP) and pulmonary vascular resistance index (PVRI) were measured by right heart catheterization. We measured the derivative of reactive oxygen molecules (d-ROMs), biological antioxidant potential (BAP) and superoxide dismutase (SOD) by automatic biochemical analyzer, malondialdehyde (MDA) and asymmetric dimethylarginine (ADMA) by enzyme-linked immunosorbent assay. The relationship between oxidative-antioxidative biomarkers and ADMA, as well as their association with pulmonary hemodynamics, were analyzed.

**Results:**

Compared with age- and gender-matched controls, there was no significant difference of d-ROMs in PAH and CTEPH patients; MDA was increased in CTEPH patients (P = 0.034); BAP and SOD were decreased in PAH (P = 0.014, P < 0.001) and CTEPH patients (P = 0.015, P < 0.001); ADMA level was significantly higher in PAH (P = 0.007) and CTEPH patients (P < 0.001). No association between oxidative-antioxidative biomarkers and ADMA was found. Serum ADMA concentration was correlated with mPAP (r = 0.762, P = 0.006) and PVRI (r = 0.603, P = 0.038) in PAH patients.

**Conclusions:**

The antioxidative potential and NO signaling are impaired in PAH and CTEPH. Increased serum ADMA level is associated with unfavorable pulmonary hemodynamics in PAH patients. Thus, ADMA may be useful in the severity evaluation and risk stratification of PAH.

## Background

Pulmonary hypertension (PH) is a complex pulmonary vascular disease caused by different reasons, such as chronic hypoxia, left heart diseases, chronic respiratory diseases and thromboembolism [1]. In the classification of PH by World Health Organization (WHO), pulmonary arterial hypertension (PAH) and chronic thromboembolic pulmonary hypertension (CTEPH) share the pathological characteristic of vascular remodeling. Pulmonary vascular remodeling is characterized by medial hypertrophy, intimal proliferative and fibrotic changes, adventitial thickening, and thrombotic lesions [1]. Although the mechanisms involved are incompletely understood, endothelial dysfunction with proliferation of endothelial cells and smooth muscle cells plays an important role in vascular remodeling [2]. One character of endothelial dysfunction is oxidative stress (OS) [3,4], which can increase the production of reactive oxygen species (ROS) and reactive nitrogen species (RNS) and decrease the bioavailable nitric oxide (NO).

All vascular cells, including endothelial cells, smooth muscle cells, and adventitial cells, can produce ROS. In patients with vascular diseases, the oxidative-antioxidative balance in the vessel wall is compromised due to the increase of ROS production by these cells, sometimes coupled with decreased antioxidant defense. ROS may trigger signals that further exacerbate smooth muscle hypercontractility, endothelial barrier dysfunction, and vascular remodeling [5], Increasing evidence demonstrates that OS plays a contributory role in the pathogenesis of PAH [6-9]. Recently, increased markers of OS have been found in various animal models, such as C57/BL6 mice exposed to hypobaric hypoxic conditions and Sprague-Dawley rats with monocrotaline-induced PAH [10,11]. However, few studies have explored the roles of oxidative-antioxidative biomarkers in PH patients.

NO is synthesized in the endothelium from L-arginine by NO synthase. Asymmetric dimethylarginine (ADMA) is an endogenous NO synthase inhibitor, which has been implicated in the pathogenesis of various cardiovascular diseases [12-16]. ADMA plasma levels are significantly elevated in idiopathic pulmonary arterial hypertension (IPAH), and correlated significantly with mixed-venous oxygen saturation, right atrial pressure, cardiac index, as well as survival [17,18].

Whether oxidative-antioxidative biomarkers and ADMA are associated with mean pulmonary artery pressure (mPAP) and pulmonary vascular resistance index (PVRI) remains unclear. Both oxidative-antioxidative imbalance and impaired NO signaling are involved in endothelial dysfunction and vascular remodeling. NO relaxes smooth muscle and decreases its metabolism, which will reduce the production of ROS. Thus, there may be some association between ADMA and oxidative-antioxidative biomarkers, which need to be investigated to further understand the pathogenesis and pathophysiologic processes of PH.

Therefore, we designed this case-control study to measure the oxidative-antioxidative biomarkers and ADMA level in the healthy controls, PAH patients, and CTEPH patients, with an attempt to identify the association between oxidative-antioxidative biomarkers and ADMA, simultaneously evaluate the association between these biomarkers and PH severity assessed by pulmonary hemodynamics. The level of these biomarkers was compared between PAH patients and their age- and gender-matched controls, between CTEPH patients and their age- and gender-matched controls respectively. The oxidative biomarkers we assessed included derivative of reactive oxygen molecules (d-ROMs) and malondialdehyde (MDA). Biological antioxidant potential (BAP) and superoxide dismutase (SOD) were assessed as the antioxidative biomarkers. d-ROMs and BAP, which are general evaluation of oxidative and antioxidative status respectively, have been used in the evaluation of disease severity and therapeutic effect in chronic obstructive pulmonary disease [19], idiopathic pulmonary fibrosis [20] and other diseases. ROS degrade polyunsaturated lipids, forming MDA, which is used as a biomarker to measure the level of OS in an organism [21,22]. SOD out-competes damaging reactions of superoxide, protects the cell from superoxide toxicity, and is widely used in scientific research and clinical practice [23,24].

## Methods

### Study population

Consecutive adult patients newly diagnosed with PAH or CTEPH by pulmonary angiography and right heart catheterization were enrolled. PH has been defined as an increase in mPAP ≥ 25 mmHg [25]. PAH is characterized by the presence of pre-capillary PH, i.e., mPAP ≥25 mmHg, pulmonary artery wedge pressure ≤15 mm Hg, and elevated pulmonary vascular resistance >3 Wood units, in the absence of other causes of pre-capillary PH. The final diagnosis of CTEPH was based on the presence of pre-capillary PH in patients with multiple chronic/organized occlusive thrombi/emboli in the elastic pulmonary arteries. Patients were excluded if they had unstable atherosclerotic vascular disease, renal dysfunction, or untreated hyperlipidemia or if they were under treatment with nitrates, NO donors, prostaglandins, endothelin receptor antagonists, sildenafil, or antioxidant therapy. The demographic and clinical information, six minute walk distance (6MWD) and WHO functional class of all the patients was recorded. mPAP and PVRI were measured by right heart catheterization.

Age- and gender-matched healthy candidates were included as the control group for PAH group and CTEPH group, respectively. Healthy controls were selected from volunteers without any abnormality in the physical examination and laboratory tests. The exclusion criteria included: 1) with a history of respiratory disease, cardiac disease, cardiovascular and cerebrovascular disease, chronic liver and kidney failure, malignancy, diabetes mellitus, and/or any additional medical disorders; 2) smoking; and 3) using antioxidant drugs.

The study protocol was approved by the Ethics Committee of Beijing Hospital (2014BJYYEC-051-01). Informed consent was obtained from each subject.

### Blood samples

Once the diagnosis was established, fresh blood samples were collected before the treatment except the basic treatment began. The basic treatment included diuretics, oxygen, cardiotonics and other supportive treatment. We used the separation gel coagulation promoting vacuum tubes. Within 1 hour after sample collection, the blood sample was centrifuged by 2280 g for 5 minutes. Then the serum was stored at -80°C. All the serum samples were measured simultaneously. Before the measurement of the biomarkers, serum samples were balanced to room temperature. Thawing and refreezing were avoided.

### Measurement of d-ROMs

We measured d-ROMs level using the automatic biochemical analyzer (HITACHI 7600 Series, Japan). The method is based on the principle that in Tris-HCl buffer (pH = 5), iron ions previously bonded to serum proteins can release and catalyze the conversion of serum hydroperoxides to alkoxyl and peroxyl radicals, which further react with chromogen, dimethyl para-phenylene diamine hydrochloride. Upon oxidation, its color becomes lighter, which is measured at 505 nm. Tert butyl hydroperoxide (TBHP) was used as standard substance. The results were expressed as an equivalent of mmol TBHP Equiv/L.

### Measurement of BAP

BAP level was measured using the automatic biochemical analyzer (AU 5400, Olympus, Japan). The assay is based on the ability of a colored ferric thiocyanate to decrease in absorption when Fe^3+^ ions are reduced to Fe^2+^. The absorbance is measured photometrically at 520 nm and calculating the amount of reduced ferric ions. Vitamin C (VitC) was used as standard substance. The results were expressed as an equivalent of mmol VitC Equiv/L.

### Measurement of SOD, MDA and ADMA

SOD level was measured using the automatic biochemical analyzer (AU 5400, Olympus, Japan) and a commercial kit (Fujian Luck Bioscience CO. LTD, China).

MDA and ADMA levels were measured by the method of enzyme-linked immunosorbent assay. Commercial kits (KYM, China) were used. The standard substance and serum samples were incubated with MDA/ADMA antibody. Subsequently, the peroxidase-conjugated anti-human IgG was bound to the anti-MDA/ADMA antibodies. After coloration for 15 min, the absorbance of each well was measured at 450 nm with an ultramark microplate reader (Bio-Rad, Hercules, CA). The concentration for each sample was calculated according to the standard curve, after subtraction of the blank values.

### Statistical analysis

SPSS software version 17.0 (Statistical Package for the Social Sciences Inc., Chicago, IL, USA) was used to analyze all the data. Kolmogorov-Smirnov method was used to test whether the data was normal distributed. Variables were presented as mean ± standard deviation, median with quartiles, or constituent ratio as appropriate. We used *t* test for two independent samples or nonparametric test to compare the continuous variable and *χ*^2^ test to compare the categorical variable between groups. Pearson Correlation was used to analyze the correlation between variables. A *P* value < 0.05 was regarded as statistically significant.

## Results

### Characteristics of study population

Totally 35 patients with PH and 35 healthy controls were enrolled. Demographic data of the patients and healthy controls were presented in Table 1. Among the PH patients, 12 patients were diagnosed with PAH (7 with idiopathic PAH, 4 with connective tissue diseases associated PAH, 1 with familial PAH), and 23 were diagnosed with CTEPH. The mean age was 44.6 years in PAH group and 55.0 years in CTEPH group. In PAH group, 2 patients (16.7%) were male. In CTEPH group, 12 patients (52.2%) patients were male. Most patients in PAH and CTEPH groups were WHO functional class II-III (74.9% and 87.0%, respectively). The comorbidity, 6MWD, level of NT-proBNP and main parameters of pulmonary haemodynamics were also presented in Table 1. The median 6MWD was 253.50 m in PAH group and 275.00 m in CTEPH group, respectively.

**Table 1:** **Characteristics of patients with PAH or CTEPH and healthy controls**

**Variables**	**PAH group (n = 12)**	**PAH-Control (n = 12)**	***P*** **value**	**CTEPH group (n = 23)**	**CTEPH-Control (n = 23)**	***P*** **value**
**Male, n (%)**	2 (16.7)	2 (16.7)	1.000	12 (52.2)	11 (47.8)	0.768
**Age, years**	44.6 ± 13.1	44.8 ± 13.2	0.963	55.0 ± 11.6	55.7 ± 11.5	0.839
**BMI, kg/m** ^**2**^	25.66 ± 4.27	25.12 ± 3.73	0.894	25.75 ± 3.23	24.53 ± 3.01	0.748
**Hypertension, n (%)**	2 (16.7)	-	-	5 (21.7)	-	-
**Diabetes Mellitus, n (%)**	0 (0)	-	-	1 (4.3)	-	-
**6MWD, m**	253.50 (148.50, 368.25)	-	-	275.00 (150.00, 419.00)	-	-
**NT-proBNP, pg/ml**	1130.0 (373.9, 2312.0)	-	-	1723.0 (609.0, 3136.0)	-	-
**mPAP, mmHg**	50 (37, 55)	-	-	52 (49, 55)	-	-
**PVRI, dyn · sec · m** ^**2**^ **/cm** ^**5**^	1398.0 (1149.0, 2647.5)	-	-	2231.5 (1595.75, 2611.5)	-	-
**CO, L/min**	3.95 ± 1.21	-	-	3.45 ± 1.19	-	-
**CI, L/min/m** ^**2**^	2.27 ± 0.67	-	-	1.82 ± 0.52	-	-

Data are presented as mean ± SD or median (interquartile range). PAH = pulmonary arterial hypertension; CTEPH = chronic thromboembolic pulmonary hypertension; BMI = body mass index; WHO = World Health Organization; 6MWD = 6-minute walk distance; NT-proBNP = N-terminal pro brain natriuretic peptide; mPAP = mean pulmonary arterial pressure; PVRI = pulmonary vascular resistance index; CO = cardiac output; CI = cardiac index.

### Comparison between PAH patients and PAH-controls

**Figure 1:**
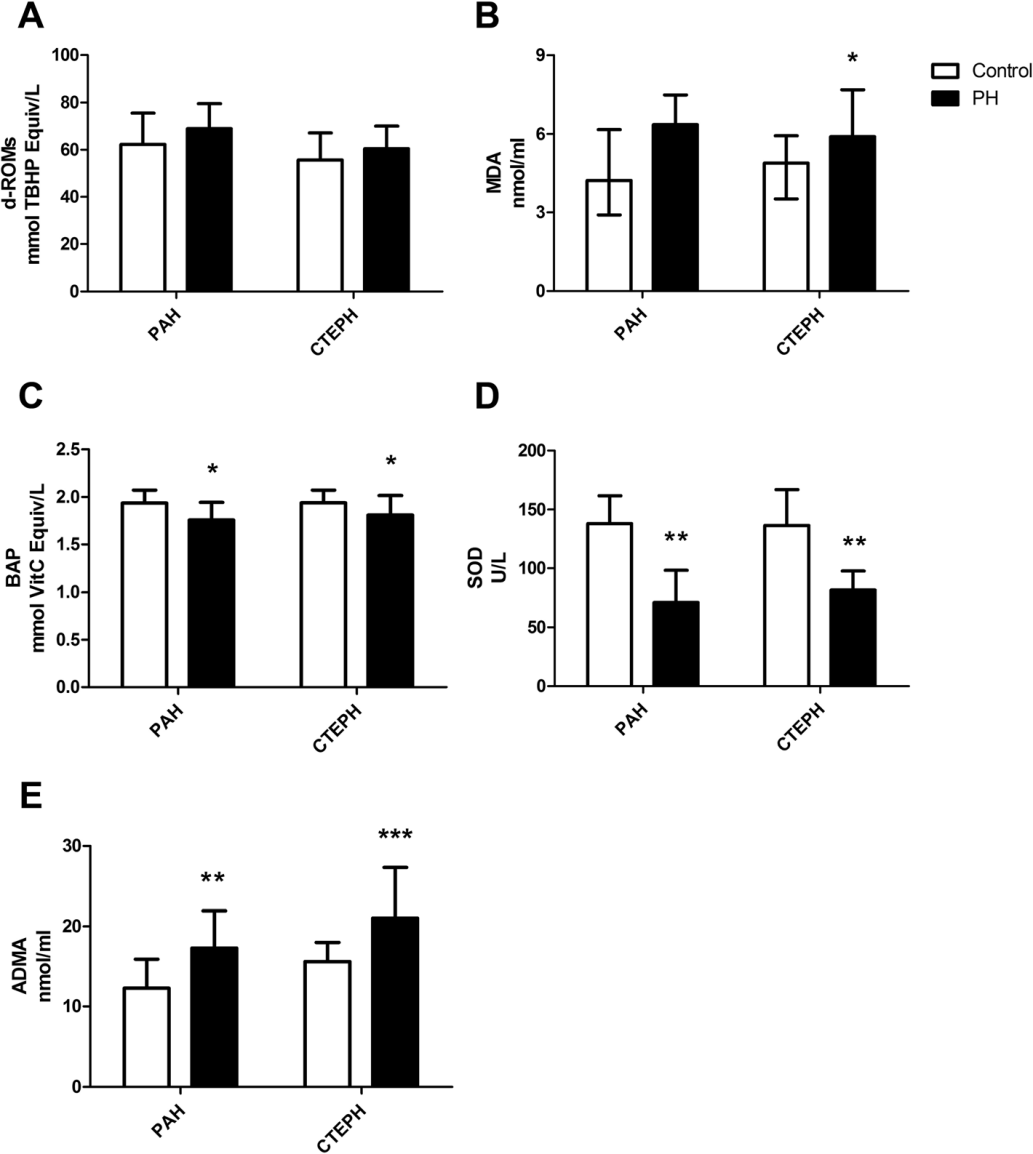
The comparison of oxidant-antioxidant biomarkers and ADMA levels. Data are presented as mean ± standard deviation in Figure **A**, **C** and **D**, presented as median (interquartile range) in Figure **B**. **A**. There was no significant difference in the level of d-ROMs between PAH group and PAH-control group, or between CTEPH and CTEPH-control group. **B**. The level of MDA was higher in PAH and CTEPH patients than that in their control group. The difference was only significant between CTEPH group and CTEPH-control group. **C**. Compared with controls, the level of BAP was significantly lower in patients with PAH or CTEPH. **D**. Compared with controls, the level of SOD was significantly lower in patients with PAH or CTEPH. **E**. Compared with controls, the level of ADMA was significantly higher in patients with PAH or CTEPH.

We compared the demographic characteristics and biomarkers level between PAH patients and PAH-controls (Table 1, Figure 1). The gender, age composition and BMI were well-matched. The mean levels of BAP and SOD were significantly lower in the PAH group compared with the control group (*P* = 0.014, *P* < 0.001, Figure 1C and D). Patients in PAH group had significantly higher ADMA level than the control group (*P* = 0.007, Figure 1E).

### Comparison between CTEPH patients and CTEPH-controls

The demographic characteristics and biomarker levels were also compared between CTEPH patients and CTEPH-controls (Table 1, Figure 1). The mean level of MDA was significantly higher in CTEPH group (*P* = 0.034, Figure 1B). The levels of BAP and SOD were obviously lower in the CTEPH group compared with the control group (*P* = 0.015, *P* < 0.001, Figure 1C and D). Patients in CTEPH group had significantly higher ADMA level than the control group (*P* < 0.001, Figure 1E).

### Correlation analysis

**Figure 2:**
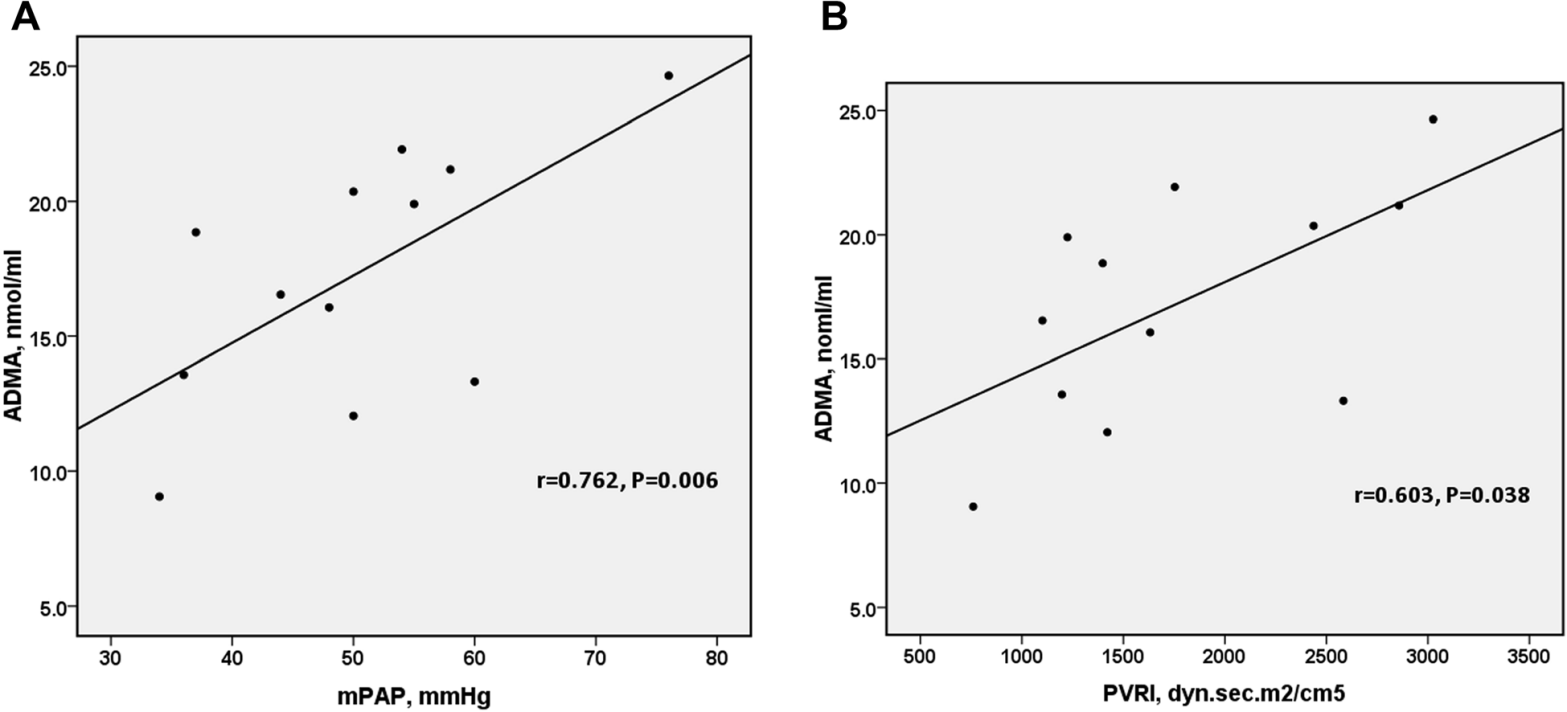
The correlation between ADMA and mPAP, PVRI in patients with PAH. In patients with PAH, mPAP (r = 0.762, *P* = 0.006) and PVRI (r = 0.603, *P* = 0.038) were both positively associated with the level of ADMA.

We analyzed the correlation between the biomarkers (including d-ROMs, MDA, BAP, SOD and ADMA) and mPAP, PVRI, 6MWD, respectively, as well as the correlations between oxidative-antioxidative biomarkers and ADMA. The Pearson correlation coefficient and *P* value were listed in Tables 2, 3, 4 and 5. No significant correlation was found between oxidative-antioxidative biomarkers and ADMA (Table 2). In patients with PAH, ADMA correlated positively with mPAP (r = 0.762, *P* = 0.006) and PVRI (r = 0.603, *P* = 0.038) (Figure 2).

**Table 2:** **The Correlation between Oxidant-antioxidant Biomarkers and ADMA**

**Biomarkers**	**PAH**		**CTEPH**	
	**r**	***P***	**r**	***P***
**d-ROMs**	-0.011	0.958	0.151	0.333
**MDA**	-0.017	0.958	0.422	0.072
**BAP**	-0.420	0.175	-0.333	0.164
**SOD**	-0.436	0.156	0.346	0.147

PAH = pulmonary arterial hypertension; CTEPH = chronic thromboembolic pulmonary hypertension; d-ROMS = derivative of reactive oxygen molecules; MDA = malondialdehyde; BAP = biological antioxidant potential; SOD = superoxide dismutase; ADMA = asymmetric dimethylarginine.

**Table 3:** **The Correlation between the Biomarkers and mPAP**

**Biomarkers**	**PAH**		**CTEPH**	
	**r**	***P***	**r**	***P***
**d-ROMs**	0.174	0.610	-0.273	0.231
**MDA**	-0.107	0.754	0.087	0.709
**BAP**	-0.460	0.155	-0.132	0.569
**SOD**	-0.341	0.305	0.118	0.611
**ADMA**	0.762	0.006*	-0.097	0.700

mPAP = mean pulmonary arterial pressure; PAH = pulmonary arterial hypertension; CTEPH = chronic thromboembolic pulmonary hypertension; d-ROMS = derivative of reactive oxygen molecules; MDA = malondialdehyde; BAP = biological antioxidant potential; SOD = superoxide dismutase; ADMA = asymmetric dimethylarginine.

*The difference with control is statistically significant.

**Table 4:** **The Correlation between the Biomarkers and PVRI**

**Biomarkers**	**PAH**		**CTEPH**	
	**r**	***P***	**r**	***P***
**d-ROMs**	0.352	0.353	-0.410	0.115
**MDA**	0.022	0.956	0.266	0.320
**BAP**	-0.581	0.101	-0.491	0.053
**SOD**	-0.654	0.056	0.043	0.875
**ADMA**	0.603	0.038*	-0.097	0.700

PVRI = pulmonary vascular resistance index; PAH = pulmonary arterial hypertension; CTEPH = chronic thromboembolic pulmonary hypertension; d-ROMS = derivative of reactive oxygen molecules; MDA = malondialdehyde; BAP = biological antioxidant potential; SOD = superoxide dismutase; ADMA = asymmetric dimethylarginine.

*The difference is statistically significant.

**Table 5:** **The Correlation between the Biomarkers and 6MWD**

**Biomarkers**	**PAH**		**CTEPH**	
	**r**	***P***	**r**	***P***
**d-ROMs**	-0.357	0.386	-0.092	0.765
**MDA**	-0.071	0.867	-0.311	0.301
**BAP**	0.546	0.162	-0.166	0.588
**SOD**	0.548	0.159	0.477	0.100
**ADMA**	-0.454	0.259	-0.369	0.264

6MWD = 6-minute walk distance; PAH = pulmonary arterial hypertension; CTEPH = chronic thromboembolic pulmonary hypertension; d-ROMS = derivative of reactive oxygen molecules; MDA = malondialdehyde; BAP = biological antioxidant potential; SOD = superoxide dismutase; ADMA = asymmetric dimethylargininenn.

## Discussion

### Oxidative-antioxidative status in PH

OS, an imbalance between ROS and antioxidant molecules, has been reported to be associated with various diseases, such as diabetes [26] and atherosclerosis [27]. Hypoxia, hypoperfusion, and instability in pulmonary circulation in PH may cause OS. In animal models, OS was found to be associated with the pathogenesis and development of PH [10]. However, few studies have explored the application of oxidative-antioxidative biomarkers in PH patients.

BAP and d-ROMs reflect the level of total antioxidants and oxidants in the samples, respectively. Previous studies found that d-ROMs level was high in COPD patients [28], and was associated with the severity of IPF [20]. BAP level was significantly lower in patients with metabolic syndrome [29], and was closely associated with diabetic retinopathy and nephropathy in patients with type 2 diabetes [30].

The most possible reason for decreased BAP and SOD level in patients with PH is the consumption of antioxidant substances in the OS. The level of d-ROMs stayed normal in PAH and CTEPH, which was possibly in compensatory status. The finding of our study suggests that OS contributes to the pathogenesis of PAH and CTEPH. In animal models, SOD augmentation regresses experimental PAH [31]. The antioxidant therapy might be used as a supplement in the treatment of PH.

In a group of 347 patients who underwent echocardiographic assessment, the relationship between pulmonary artery systolic pressure (PASP) measured by echocardiography and plasma aminothiol OS markers was investigated. For each 1% increase in plasma cystine, PASP increased by 16% [32]. However, in our current study, we did not find correlation between oxidative-antioxidative biomarkers and mPAP or PVRI measured in right heart catheterization. One possible reason is that we used different OS biomarkers and different procedures to measure pulmonary artery pressure. Another possible reason is the limited sample size of our study. Right heart catheterization is the golden standard for PH diagnosis and mPAP is more accurate than PASP estimated in echocardiography. The relationship between OS biomarkers and pulmonary artery pressure needs to be explored in more patients who undergo right heart catheterization assessment.

### Increased ADMA in PH and its significance

ADMA is a natural ubiquitous amino acid. The lungs are a major source of NO synthase and ADMA, which are in a dynamic balance and determine NO production [33]. ADMA has multiple functions, such as vasoconstriction, impairing endothelial-related dilatation, and increasing endothelial adhesivity [34]. Plasma ADMA levels are related with endothelial dysfunction. ADMA levels are elevated in many cardiovascular and metabolic diseases such as coronary artery disease [35] and hypertension [36]. In our study, ADMA level was elevated in PH, which is consistent with previous studies [17,18,37-39]. The potential mechanism that ADMA contributes to PH has been studied recently. ADMA is metabolized by dimethyl-arginine dimethylaminohydrolase. Suppression of endothelial dimethyl-arginine dimethylaminohydrolase expression and function represents as an important underlying mechanism in hypoxia-induced PH [40] and IPAH [41]. ADMA may induce pulmonary endothelial dysfunction via changes in the expression and activity of connexin 43 [42].

Also in our current study, the serum ADMA level correlated positively with mPAP in PAH patients, as well as PVRI. In previous studies, plasma ADMA concentrations correlated with mixed venous saturation, right atrial pressure, and cardiac index [18]. These findings indicate that the down-regulation of NO/cGMP pathway plays a crucial role in PAH, and the inhibition degree of this pathway affects the disease severity. The possible explanation for this association is that the NO/cGMP pathway regulates pulmonary vascular tone, which can be reflected directly by mPAP and PVRI. The pathophysiological role of ADMA in PAH still remains to be investigated. The relationship between the ADMA and NO system is probably more complicated than currently known. Since ADMA correlates with multiple parameters of pulmonary hemodynamics, it may be useful in the evaluation of disease severity, assessment of therapeutic efficiency, and risk stratification in patients with PAH or other kinds of PH. Given the small sample size of our study, further studies are needed to explore the values of ADMA in PH.

### Relationship between oxidative-antioxidative biomarkers and ADMA

Accumulating evidences have demonstrated that NO is closely associated with ROS. During chronic hypoxia, increased vascular superoxide anion production adversely impact endothelial function by impairing NO signaling [43]. When enough ROS are produced, they will start reacting readily with NO to form the peroxynitrite [44]. NO, the endothelium-derived relaxing factor, decreases smooth muscle metabolism and reduces ROS production. ADMA increases intracellular ROS generation in bovine retinal capillary endothelial cells [45]. In this study, we did not find association between oxidative-antioxidative biomarkers and ADMA in peripheral serum. The level of these biomarkers in peripheral circulation is a reflection of overall production and cleavage of ROS, RNS, and NO, which is more complicated and may be affected by various factors.

The pathogenesis of PH is a complex multifactorial process. OS and impaired NO/sGC/cGMP signal pathway are both involved, and their relationship is complicated. We speculate that ROS increase first, then NO decreases. The three major stimuli that drive the vascular remodeling process are inflammation, shear stress and hypoxia. All these stimuli may increase the production of ROS, which leads to low levels of tetrahydrobiopterin and L-arginine, the rate limiting co-factor and substrate for endothelial NO synthase. Then endothelial NO synthase is uncoupled, resulting in decreased NO production and increased ROS production in turn [9]. We think that this process happens rapidly in our body, therefore, we couldn’t detect the change of oxidative-antioxidative biomarkers before the change of NO signaling biomarkers like ADMA and vice versa.

## Conclusions

In summary, the antioxidative potential decreases while serum ADMA is elevated in PAH and CTEPH patients. Increased ADMA serum levels are associated with unfavorable pulmonary hemodynamics in PAH patients. ADMA may be useful in the severity evaluation and risk stratification of PAH.

### Ethics statement

The study protocol was approved by the Ethics Committee of Beijing Hospital (2014BJYYEC-051-01). Informed consent was obtained from each subject.

## Abbreviations

OS: Oxidative stress
NO: Nitric oxide
PH: Pulmonary hypertension
PAH: Pulmonary arterial hypertension
CTEPH: Chronic thromboembolic pulmonary hypertension
mPAP: Mean pulmonary artery pressure
PVRI: Pulmonary vascular resistance index
d-ROMs: Derivative of reactive oxygen molecules
BAP: Biological antioxidant potential
SOD: Superoxide dismutase
MDA: Malondialdehyde
ADMA: Asymmetric dimethylarginine
WHO: World Health Organization
OS: Oxidative stress
ROS: Reactive oxygen species
RNS: Reactive nitrogen species
IPAH: Idiopathic pulmonary arterial hypertension
6MWD: Six minute walk distance
TBHP: Tert butyl hydroperoxide
VitC: Vitamin C
PASP: Pulmonary artery systolic pressure
